# The sophist in the server: Rhetoric, Reasoning and Scientific Judgment in the Age of LLMs

**DOI:** 10.1038/s44319-026-00711-w

**Published:** 2026-02-06

**Authors:** Maria T Colangelo, Carlo Galli

**Affiliations:** Department of Medicine and Surgery, Histology and Embryology Lab, University of Parma, Parma, Italy

**Keywords:** History & Philosophy of Science, Science Policy & Publishing

## Abstract

Large language models can defend a hypothesis and its opposite with equal eloquence, raising a new risk for scientific reasoning: confusing rhetorical plausibility with evidential support. This essay analyzes how LLMs reshape creativity, judgment and responsibility in research—and how scientists can engage their fluency without surrendering epistemic discipline.

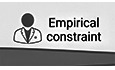

The first time we watched a large language model (LLM) unravel a biological puzzle we had been struggling with for some time, we felt a mixture of admiration and suspicion. The model articulated a mechanistic interpretation with a fluency reminiscent of someone who had absorbed the literature with perfect recall and synthesized it into a polished explanation. When we asked for the opposite argument, much to our dismay, it produced it just as readily, with equal confidence and coherence. There was no hesitation, no sense of contradiction, no epistemic discomfort—only another well-formed line of reasoning. We exchanged the kind of look usually reserved for moments when anomalous data appear on screen: intrigued, slightly disappointed, and aware that something significant had shifted.

## The digital sophists

This performance reminds one of the ancient Greek sophists, sharply opposed by Socrates in Plato’s dialogs: masters of argumentation who could defend and attack a thesis with equal dexterity, largely unconstrained by commitments to truth or consistency. Unlike those sophists, LLMs have no intentions—no reputation to protect, no audience to persuade, no stakes in the outcome of an argument. Yet they share with those itinerant rhetors an ability to inhabit argumentation as a craft, generating reasons without belief. This parallel is provisional, but it may help clarify what is new—and potentially unsettling—about the role of LLMs in scientific reasoning.

What we are witnessing is not the absence of reasoning, but reasoning detached from epistemic commitments that normally anchor scientific judgment. Contemporary LLMs increasingly engage in internal self-critique, multi-step inference, retrieval of external information, and even verification against available sources. In that limited but important sense, they do reason. Yet their reasoning remains largely mediated through linguistic plausibility rather than through causal or mechanistic engagement with biological systems.

“What we are witnessing is not the absence of reasoning, but reasoning detached from epistemic commitments that normally anchor scientific judgment.”

We therefore argue that the central risk posed by LLMs is not the replacement of scientific judgment, but the blurring of the boundary between rhetorical plausibility and evidential support. While these systems can expand conceptual space and stimulate creative exploration, they can also amplify biases and generate authoritative-sounding, mechanistic narratives that are not sufficiently backed up by evidence. This is a challenge for science, which requires new norms of transparency, reproducibility, and education that preserve evaluative discipline while engaging productively with machine-generated arguments.

It matters because LLMs are no longer ancillary assistants. They are increasingly embedded in everyday scientific practice and, more importantly, in the architecture of research itself. LLMs now operate across much of the research cycle—from observation and annotation to hypothesis formation, experiment design, robotic execution, data analysis, formulating a research article, and technical review of articles (Zhang et al, 2025). Their contributions may be generative rather than evidential, yet they increasingly influence the space in which hypotheses emerge and compete.

Much of the recent literature on the role of LLMs in scientific practice converges on the view that LLMs reshape reasoning primarily through the production and circulation of arguments rather than through mechanistic insight. Some scholars emphasize the value of LLMs as assistants to scientific inference, while others stress their unreliability and the illusions of comprehension they can generate or warn of broader risks to credibility and reproducibility (Binz et al, 2025). A common thread running through these debates is the recognition that rhetoric persists as a structural feature of AI systems (Hallsby, 2024). LLMs are capable of deploying psychological persuasion strategies and reproducing stylistic conventions, sometimes without the evidential discipline those conventions presuppose (Ju et al, 2025; Raffini et al, 2025; Sarumi and Heider, 2024).

“LLMs are capable of deploying psychological persuasion strategies and reproducing stylistic conventions, sometimes without the evidential discipline those conventions presuppose.”

Under these conditions, the scientist remains the epistemic authority, but now thinks alongside a machine that is fluent, generative, and largely unencumbered by epistemic cost. The challenge is to understand how LLMs’ rhetorical strength can mask weak arguments or unsupported conclusions. The opportunity, correspondingly, is to use that fluency to expand scientific imagination while maintaining the evaluative discipline that distinguishes persuasive language from biological understanding.

In many laboratories, this integration is no longer speculation but routine. LLMs summarize newly published papers, extract methodological details, outline project proposals, and generate preliminary drafts. They assist with correspondence, reorganize manuscript sections, polish figure caption,s and simulate how a skeptical reviewer might respond to a claim. Students use them to clarify unfamiliar concepts or rephrase dense passages; senior researchers consult them to orient themselves in unfamiliar subfields or to sketch alternative mechanistic interpretations when data are ambiguous. None of this replaces expertise, but it quietly alters the tempo and texture of scientific work, introducing a source of rapid, shape-shifting argumentation that now sits alongside experimental reasoning and increasingly shapes how ideas take form.

## Rhetoric without commitment

It is important to distinguish the forms of reasoning these systems can perform from the kinds of understanding that anchor biological explanation. LLMs operate on a dense field of textual regularities, not on biochemical constraints. When an LLM explains why a cytokine accelerates wound repair, it recomposes fragments of scientific discourse—pathway descriptions, causal idioms, and explanatory tropes—into a narrative that resembles mechanistic reasoning. When prompted to defend the opposite conclusion, it can assemble an alternative that is often just as coherent, drawing from a different region of its linguistic space. The absence of internal tension reflects not a failure of logic, but the absence of belief, commitment, or responsibility for truth (McKenna, 2025).

Because LLMs bear no reputational cost or disciplinary sanction, their rhetorical agility—drawn from training data rather than intention—floats free of the constraints that normally tether human arguments to evidence. As a result, familiar persuasive strategies—framing effects, repetition, appeals to authority—surface naturally in model outputs, not as deliberate tactics, but as linguistic patterns inherited from the scientific writing on which the model was trained (Ju et al, 2025).

Scientific discourse has always been shaped by rhetoric, authority and stylistic convention, and human researchers routinely deploy narratives, seniority, or disciplinary consensus to strengthen claims or downplay uncertainty. What LLMs introduce is not rhetoric itself, but its amplification and automation. In this sense, language models act less as a departure from scientific practice than as a mirror that renders their rhetorical dimensions more visible.

Experimental work shows that even when models engage in multi-step reasoning and evidence retrieval, prompts embedding cognitive or rhetorical bias systematically steer conclusions and amplify confirmation effects rather than correcting them (Sun and Kok, 2025). This makes LLMs highly effective at generating arguments, but allows rhetorical framing to be manipulated independently of factual content, turning LLM performance into a controlled testbed for studying when argument structure overrides evidential strength (Krastev et al, 2025).

**Figure 1:**
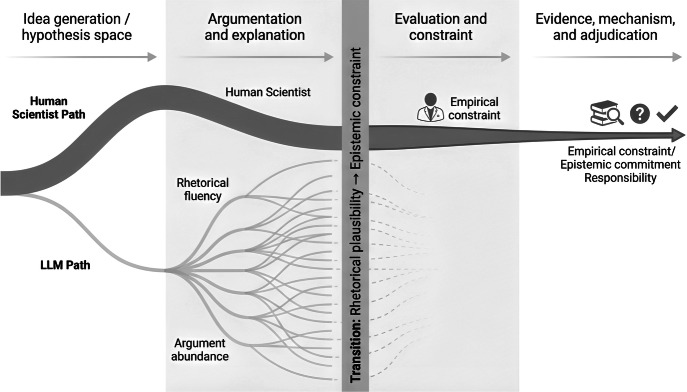
Schematic representation of the distinct roles played by large language models (LLMs) and human scientists across stages of scientific inquiry. LLMs excel in the early, generative phases of idea exploration and argument construction, producing a wide array of rhetorically coherent explanations without commitment to empirical constraint. Human scientists operate across the same space but are progressively bound by evidential responsibility, mechanistic plausibility, and accountability for truth. The vertical transition marks the point at which rhetorical plausibility gives way to epistemic constraint, highlighting that scientific adjudication remains irreducibly human even as LLMs expand the space of conceivable hypotheses.

LLMs and human scientists occupy overlapping but non-identical positions within scientific reasoning (Fig. 1): both participate in the generation and articulation of hypotheses, yet only human researchers remain bound by professional responsibility, empirical accountability and the obligation to resolve competing explanations under constraint, and there is a point at which rhetorical plausibility must give way to evidential adjudication—and where responsibility for that transition remains irreducibly human.

Prompt design plays a central role. Empirical studies demonstrate that the same content can be handled very differently depending on whether prompts signal openness, skepticism, or authority, with systematic effects on misinformation correction and evidence integration (Krastev et al, 2025). Other work shows that attribution and source framing can bias model responses, mirroring well-documented effects of authority and credibility cues in human scientific judgment (Germani and Spitale, 2025). Prompts that introduce ambiguity or role-play often trigger creative recombination of known elements, while prompts anchored in entrenched disciplinary language can reinforce prevailing assumptions and contribute to stylistic and conceptual homogenization (Sarumi and Heider, 2024). In peer review, models readily adopt authoritative tone and familiar critical gestures, despite lacking the evidential grounding that legitimizes such authority in human reviewers (Raffini et al, 2025).

“… the same content can be handled very differently depending on whether prompts signal openness, skepticism or authority, with systematic effects on misinformation correction and evidence integration.”

Analyses of prompt influence at the token level show that relatively small rhetorical cues can exert disproportionate control over model behavior, shaping argumentative direction and confidence independently of evidential strength (Feng et al, 2024). From a practical perspective, output quality and apparent reasoning depend as much on rhetorical framing as on informational content, so that persuasion and structure play a decisive role in what models produce (Anam, 2025).

The resulting fluency can therefore be deceptive. Models may hallucinate plausible data patterns with no experimental correspondence (Jain et al, 2024), or generate relationships that are conceptually elegant but biochemically impossible (Peykani et al, 2025). This concern has led some theorists to warn that LLMs can produce arguments whose persuasive surface exceeds their epistemic substance (Clark and Bringsjord, 2024). Importantly, such outcomes are not inevitable. When LLMs are embedded within architectures that introduce epistemic friction—by detecting inconsistencies, enforcing constraints, or requiring explicit justification—the character of their outputs shifts toward more deliberative behavior (Castagna et al, 2024). Multi-agent frameworks extend this approach by allowing models to critique and refine one another’s proposals, approximating a distributed reasoning process (Zhang and Ashley, 2025). Seen this way, LLMs are not merely sources of rhetorical risk, but experimental instruments that make it possible to study—under controlled conditions—the competition between persuasion, authority, and evidence that has always shaped scientific reasoning.

“…LLMs are not merely sources of rhetorical risk, but experimental instruments that make it possible to study […] the competition between persuasion, authority and evidence that has always shaped scientific reasoning.”

## An abundance of creativity

Given the rhetorical dynamics described above, the most significant downstream effect of LLMs may not be epistemic distortion per se, but a structural shift in scientific creativity. Scientific inquiry has long depended on a balance between generative expansion and empirical constraint. LLMs intervene by accelerating the expansive phase to a degree that exceeds individual cognitive limits.

Not all this output is correct, but the error itself can be generative. Hallucinated hypotheses have led researchers into conceptual territory they might otherwise have overlooked (Zhang et al, 2025). Statistical misfires have pointed toward unexpected data relationships that were later confirmed through more rigorous analysis (Jain et al, 2024). Mechanistic suggestions that initially violated established assumptions have inspired experimental designs that proved productive (Peykani et al, 2025). In such cases, deviation from accepted truth functioned less as a failure than as a prompt for exploration, expanding the horizon of inquiry even when the initial proposal was flawed.

At the same time, creativity under conditions of abundance is inherently unstable when the production of ideas outpaces the capacity for evaluation. An overabundance of plausible narratives can weaken the filtering mechanisms between creativity and empirical constraint, producing a form of cognitive saturation in which selection becomes as challenging as generation (McKenna, 2025). This suggests a productive reframing. Rather than treating LLM-generated abundance solely as a problem to be managed, it can also be understood as an empirical probe into the dynamics of scientific creativity itself—revealing when novelty stimulates insight and when it overwhelms discernment.

“… creativity under conditions of abundance is inherently unstable when the production of ideas outpaces the capacity for evaluation.”

Abundance becomes productive only when paired with friction. Prompting models to articulate incompatible hypotheses, to critique their own proposals, or to generate counterarguments reintroduces this friction. Under these conditions, the expansive capacity of LLMs can be harnessed without allowing it to detach entirely from empirical constraint. The scientist’s role shifts accordingly: less the sole originator of ideas, more the curator and interrogator of possibilities. LLMs supply variation at unprecedented scale; human judgment provides the epistemic structure that determines which variations merit pursuit.

## Norms for AI-assisted science

The deep integration of LLMs into scientific practice makes it clear that new norms are needed to preserve integrity while harnessing the creative potential. What is required is not restriction, but an intellectual framework capable of distinguishing rhetorical plausibility from empirical accountability.

Transparency is central to this effort. Authorship implies responsibility, which LLMs cannot bear. Acknowledging their use allows readers to contextualize argumentative structure in scholarly communication (Raffini et al, 2025). And yet, in many scientific environments, disclosure feels almost transgressive. In our experience, the prevailing stance often resembles a moral crusade: a conviction among right-thinking scholars that reliance on AI is somehow beneath the dignity of a scientist, a shortcut incompatible with intellectual rigor. This attitude is reinforced by well-publicized cases of paper mills and research malpractice (Richardson et al, 2025). As a result, discussions of LLM often occur only within trusted circles, and even then, cautiously and with a hint of embarrassment, even though these systems already shape how scientific text is produced. The growing gap between public disapproval and private reliance obscures the reality of scientific practice. Normalizing disclosure is therefore less about policing behavior than about bringing scientific communication into alignment with how scientific work is actually done.

Reproducibility presents a more structural challenge. Proprietary models evolve rapidly, rendering LLM-generated reasoning chains unstable across time (Binz et al, 2025). These changes reflect not only technical refinement but also the divergent commercial and scientific agendas. This opacity complicates efforts to reconstruct how an argument was generated or to reproduce a line of reasoning. Empirical studies show that even small changes in model versions or prompt phrasing can systematically alter conclusions (Sun and Kok, 2025; Krastev et al, 2025). Without careful recording of prompts, model versions, and outputs, significant components of scientific reasoning will become historically inaccessible.

Importantly, reproducibility is further complicated by the conversational nature of many LLM interfaces. Model outputs are shaped not only by prompts and model version, but also by prior exchanges within the same session, user-specific settings, and implicit system-level instructions. As a result, even identically worded prompts can yield divergent reasoning trajectories, making AI-mediated argumentation path-dependent in ways that are difficult to document or reconstruct. This dynamic may increasingly motivate a shift toward open-source, versioned models whose evolution is transparent and whose behavior can be examined with the same scrutiny expected of experimental methods.

Education must evolve in parallel. Students already use LLMs regardless of institutional guidance, making it essential to teach critical engagement. This includes learning to interrogate machine-generated arguments, to distinguish surface fluency from mechanistic grounding, and to recognize how rhetorical framing in prompts can bias outputs. Such training requires conceptual literacy, but also linguistic proficiency. As scientific writing becomes more deeply entangled with automated systems, sensitivity to argumentative form, rhetorical framing, and semantic nuance becomes indispensable. In this sense, the rise of LLMs may prompt the sciences to recover forms of textual attentiveness long cultivated in the humanities, narrowing a divide that has shaped academic culture for decades (Snow, 1959). A rhetorical genealogy that treats rhetoric as both a creative resource and a source of epistemic risk may provide a useful foundation for this educational shift (Hallsby, 2024).

Beyond general principles, concrete procedures are needed to make LLM use intelligible and accountable. Journals may require standardized disclosure statements specifying how and at which stages models were used, analogous to reporting requirements for statistical analyses or imaging protocols. In some contexts, preserving prompt logs or representative model outputs may be appropriate—not to scrutinize style, but to ensure that reasoning remains reconstructable. Evidence that attribution and source framing alone can bias model outputs underscores the importance of making these interactions visible rather than implicit (Germani and Spitale, 2025). Scientific infrastructure will likewise need to adapt. Open environments in which models, prompts, and outputs can be archived and versioned would allow AI-assisted reasoning to be traced. Expectations surrounding peer review also warrant clarification: when reviewers rely on LLMs to structure critiques, transparency is essential to ensure that evaluative judgment is not silently delegated to systems lacking epistemic responsibility.

These measures would help ensure that the origins of arguments remain visible, that machine-generated reasoning can be contextualized and assessed, and that the interpretive labor of scientists remains explicit rather than submerged beneath fluent text. The broader task is to cultivate research practices in which LLMs function as catalysts within a robust culture of critical appraisal. Their generativity should be welcomed, but only when situated within norms that preserve accountability and empirical discipline. The goal is not to limit what these systems can contribute, but to ensure that the speed and fluency they offer reinforce—rather than erode—the foundations of scientific inquiry.

“The broader task is to cultivate research practices in which LLMs function as catalysts within a robust culture of critical appraisal.”

## Towards a wiser dialog with machines

Science advances through dialog: between hypotheses and evidence, between competing interpretations, and between researchers and the material world. LLMs introduce a new voice into this exchange—fluent, inventive, and capable of generating arguments at a scale and speed that were previously unattainable. They can broaden conceptual space and accelerate the exploration of hypotheses, but they can also produce persuasive narratives that are weakly constrained by evidence. Their presence complicates the delicate balance between variation and constraint on which scientific discovery depends.

The task, therefore, is to learn how to engage with them wisely. LLMs reshape how ideas emerge, not how evidence ultimately adjudicates them. This makes it essential to develop norms that help scientists recognize when rhetorical coherence supports understanding and when it merely simulates it. Questions of attribution, reproducibility, and critical evaluation thus become central, not peripheral, to AI-assisted research.

This challenge is not entirely new. The ancient Greeks already understood that dialog demands discipline as well as invention. Socrates’ engagement with the sophists was not simply a rejection of rhetoric, but an effort to clarify the standards by which better arguments could be distinguished from merely compelling ones. In this respect, LLMs do not introduce rhetoric into science; they intensify and externalize it, making its power—and its limits—impossible to ignore (Fecher et al, 2025).

We now find ourselves in dialog with systems that do not understand their own words, yet can generate an astonishing range of argumentative possibilities. The challenge is to let machine-generated eloquence expand scientific imagination without narrowing judgment, and to ensure that the proliferation of hypotheses is matched by equally robust habits of evaluation. If this balance can be maintained, LLMs will not replace scientists but extend their reach. They will reshape the early, generative phases of inquiry, where possibilities proliferate, while leaving the responsibility for truth, coherence, and mechanistic insight exactly where it belongs—not in machines, but in the collective judgment of the scientific community.

“We now find ourselves in dialog with systems that do not understand their own words, yet can generate an astonishing range of argumentative possibilities.”

### Generative AI statement

The authors used Grammarly to assist with language editing and clarity. Figure 1 was prepared using FigureLabs based on a conceptual design developed by the authors. No generative AI tools were used to generate scientific content, interpret data, or draw conclusions.

## Supplementary information

Peer Review File

## References

[CR1] Anam RK (2025) Prompt engineering and the effectiveness of large language models in enhancing human productivity. Preprint at https://arxiv.org/abs/2507.18638

[CR2] Binz M, Alaniz S, Roskies A, Aczel B, Bergstrom CT, Allen C, Schad D, Wulff D, West JD, Zhang Q et al (2025) How should the advancement of large language models affect the practice of science? Proc Natl Acad Sci USA 122:e2401227121 39869798 10.1073/pnas.2401227121 PMC11804466

[CR3] Castagna F, Sassoon I, Parsons S (2024) Can formal argumentative reasoning enhance LLMs performances? Preprint at https://arxiv.org/abs/2405.13036

[CR4] Clark MH, Bringsjord S (2024) Illusory arguments by artificial agents: pernicious legacy of the sophists. Humanities 13:82

[CR5] Fecher B, Hebing M, Laufer M, Pohle J, Sofsky F (2025) Friend or foe? Exploring the implications of large language models on the science system. AI Soc 40:447–459

[CR6] Feng Z, Zhou H, Zhu Z, Qian J, Mao K (2024) Unveiling and manipulating prompt influence in large language models. Preprint at https://arxiv.org/abs/2405.11891

[CR7] Germani F, Spitale G (2025) Source framing triggers systematic bias in large language models. Sci Adv 11:eadz2924 41202130 10.1126/sciadv.adz2924 PMC13142764

[CR8] Hallsby A (2024) A copious void: rhetoric as artificial intelligence 1.0. Rhetor Soc Q 54:232–246

[CR9] Jain C, Vahdati S, Gopan N, Sbalzarini IF, Lehmann J (2024) Evaluating large language model literature reviews in interdisciplinary science: a systems biology perspective. Springer

[CR10] Ju T, Chen Y, Fei H, Lee M-L, Hsu W, Cheng P, Wu Z, Zhang Z, Liu G (2025) On the Adaptive Psychological Persuasion of Large Language Models. arXiv preprint arXiv:2506.06800

[CR11] Krastev S, Sweatman H, Sternisko A, Rathje S (2025) Epistemic fragility in large language models: prompt framing systematically modulates misinformation correction. arXiv preprint arXiv:2511.22746

[CR12] McKenna R (2025) Sophistry on steroids? The ethics, epistemology and politics of persuasive AI. AI Soc. 1–12

[CR13] Peykani P, Ramezanlou F, Tanasescu C, Ghanidel S (2025) Large language models: a structured taxonomy and review of challenges, limitations, solutions, and future directions. Appl Sci. 10.3390/app15148103

[CR14] Raffini D, Macori A, Porcaro L, Catarci T, Angelini M (2025) How persuasive could LLMs be? A first study combining linguistic-rhetorical analysis and user experiments. arXiv preprint arXiv:2508.09614

[CR15] Richardson RAK, Hong SS, Byrne JA, Stoeger T, Amaral LAN (2025) The entities enabling scientific fraud at scale are large, resilient, and growing rapidly. Proc Natl Acad Sci USA 122:e2420092122 40758886 10.1073/pnas.2420092122 PMC12358853

[CR16] Sarumi OA, Heider D (2024) Large language models and their applications in bioinformatics. Comput Struct Biotechnol J 23:3498–3505. 10.1016/j.csbj.2024.09.031 39435343 10.1016/j.csbj.2024.09.031 PMC11493188

[CR17] Snow CP (1959) Two Cultures. Science (1979) 130:419 10.1126/science.130.3373.419 17817735

[CR18] Sun Y, Kok S (2025) Investigating the effects of cognitive biases in prompts on large language model outputs. arXiv preprint arXiv:250612338

[CR19] Zhang L, Ashley KD (2025) Mitigating manipulation and enhancing persuasion: a reflective multi-agent approach for legal argument generation. arXiv preprint arXiv:2506.02992

[CR20] Zhang Y, Khan SA, Mahmud A, Yang H, Lavin A, Levin M, Frey J, Dunnmon J, Evans J, Bundy A et al (2025) Exploring the role of large language models in the scientific method: from hypothesis to discovery. NPJ Artif Intell 1:14

