# Supplementary material for: The sophist in the server: Rhetoric, Reasoning and Scientific Judgment in the Age of LLMs
Source: EMBO Rep. 2026 Feb 6;27(5):1098–102. doi: 10.1038/s44319-026-00711-w (PMC12979577; doi:10.1038/s44319-026-00711-w)
Supplement: Supplementary file 1 — Peer Review File [file 44319_2026_711_MOESM1_ESM.pdf]

EMBO reports

The Sophist in the Server

Maria Colangelo and Carlo Galli

Corresponding author(s): Carlo Galli (carlo.galli@unipr.it)

Review Timeline:

Submission Date:	11th Dec 25
Editorial Decision:	19th Dec 25
Revision Received:	1st Jan 26
Accepted:	29th Jan 26

Editor: Holger Breithaupt

Transaction Report: This article was editorially reviewed.
